# Purification and Characterization of a White Laccase with Pronounced Dye Decolorizing Ability and HIV-1 Reverse Transcriptase Inhibitory Activity from *Lepista nuda*

**DOI:** 10.3390/molecules21040415

**Published:** 2016-03-26

**Authors:** Mengjuan Zhu, Guoqing Zhang, Li Meng, Hexiang Wang, Kexiang Gao, Tb Ng

**Affiliations:** 1 Department of Fungal Resource, College of Plant Protection, Shandong Agricultural University, 61, Daizong Street, Tai’an 271018, China; mengfan7777@163.com (M.Z.); mengli0121@126.com (L.M.); 2 State Key Laboratory for Agrobiotechnology, Department of Microbiology, China Agricultural University, Beijing 100193, China; 3 Key Laboratory of Urban Agriculture (North) of Ministry of Agriculture, College of Biological Science and Engineering, Beijing University of Agriculture, Beijing 102206, China; zhanggqbua@163.com; 4 School of Biomedical Sciences, Faculty of Medicine, The Chinese University of Hong Kong, Shatin, New Territories, Hong Kong, China

**Keywords:** laccase, purification, sequence, dye decolorizing ability, HIV-1 reverse transcriptase inhibitory activity

## Abstract

A strain LN07 with high laccase yield was identified as basidiomycete fungus *Lepista nuda* from which a white laccase without type I copper was purified and characterized. The laccase was a monomeric protein with a molecular mass of 56 kDa. Its N-terminal amino acid sequence was AIGPAADLHIVNKDISPDGF. Besides, eight inner peptide sequences were determined and lac4, lac5 and lac6 sequences were in the Cu^2+^ combination and conservation zones of laccases. HIV-1 reverse transcriptase was inhibited by the laccase with a half-inhibitory concentration of 0.65 μM. Cu^2+^ ions (1.5 mM) enhanced the laccase production and the optimal pH and temperature of the laccase were pH 3.0 and 50 °C, respectively. The *Km* and V*max* of the laccase using ABTS as substrate were respectively 0.19 mM and 195 μM. Several dyes including laboratory dyes and textile dyes used in this study, such as Methyl red, Coomassie brilliant blue, Reactive brilliant blue and so on, were decolorized in different degrees by the purified laccase. By LC-MS analysis, Methyl red was structurally degraded by the laccase. Moreover, the laccase affected the absorbance at the maximum wavelength of many pesticides. Thus, the white laccase had potential commercial value for textile finishing and wastewater treatment.

## 1. Introduction

Laccases (EC 1.10.3.2) constitute a group of multicopper and polyphenol oxidases, which catalyze the oxidation of a great diversity of organic aromatic substrates concomitantly with the reduction of molecular oxygen to water [1]. It was first discovered in exudates of the lacquer tree *Rhus vernicifera* by Yoshida H in 1883 and has subsequently been detected in a variety of organisms, such as fungi, plants, bacteria, and some insects [2,3]. However, most of the laccases studied so far originated from fungal species and the laccase activity has been demonstrated in Basidiomycetes, Ascomycetes and Deuteromycetes [4,5]. In fungi, laccases have more diverse physiological functions such as sporulation, pigment production, fruit body formation and plant pathogenesis [6,7,8].

Most fungal laccases are extracellular monomeric globular proteins with a molecular mass of approximately 50–100 kDa with an acidic isoelectric point around pH 4.0. They are generally glycosylated, with an extent of glycosylation mostly ranging from 10% to 25% and higher than 30% only in a few cases [2,8,9,10,11,12]. Based on multiple sequence alignments of more than 100 laccases, a set of four ungapped sequence regions was found to identify the laccases and to distinguish them within the broader class of blue multicopper oxidases [13]. Generally speaking, laccases contain four copper atoms which are divided into three types (type I, type II and type III) according to their spectroscopic properties and the four copper atoms are positioned in the active center that can be turned into a unique structure of laccases’ catalytic center [10,14]. Type I copper with one Cu^2+^ ion has an absorbance at 614 nm mainly accounts for the typical blue color of laccases [15] and the enzymes lacking the copper atom responsible for the blue color are called “yellow” or “white” laccases [16,17]. Type II copper with one Cu^2+^ ion and type III copper with two Cu^2+^ ions are closely arranged in a trinuclear cluster, but different in structure and functions [2,10,18].

Laccases have been intensively studied recently due to their attractiveness for dozens of biotechnological applications in several areas, such as textile industry, paper and pulp industries, food, organic synthesis, environmental, pharmaceutical, bioremediation, biosensor, and so on [5,8,12]. The application in dealing with the effluents generated from textile industry is the most extensively reported [19,20,21]. Water from textile industries polluted with dyes is reported to be one of the top ten contaminating sources of water bodies [20]. The chemical structures of dyes used in dyeing textiles such as triarylmethane, indigoid, azo and athraquinonic dyes provide a resistance to fading when exposed to light, water and other chemicals [22,23]. Traditional processes either cannot remove all dyes or are costly but laccases may provide a green and efficient alternative for decolorizing dyes and even detoxification before discharge into the environment. This is why laccase-based processes are being used in industry nowadays [22,24,25]. During the decolorization, suitable compounds called mediators acting as intermediate substrates can enable laccases to indirectly oxidize large molecules and even nonphenolic substrates. The first mediator used in the laccase-mediator system (LMS) was 2,7-azinobis(3-ethylbenzothia-zolone-6-sulfonic acid) diammonium salt (ABTS) [2,26]. Another application of laccases which has aroused the interest of researchers is the degradation of pesticides. Due to extensive use of pesticides for better agricultural productivity, contamination of soil and water takes place resulting a serious environmental problem [4]. Several pesticides such as polychlorinated biphenyls, xylene, polycyclic aromatic hydrocarbons, pentachlorophenol, and trinitrotoluene are known for their carcinogenic as well as mutagenic effect and seriously affect human health. Many pesticides are the substrates of laccases, thus laccases can remove a wide variety of hazardous chemicals, which is the reason why researchers are interested in laccases [22,27,28].

*Lepista nuda*, widespread in China, is an edible mushroom with lavender-colored cap and lamella. Until now, only one metalloprotease has been purified from this mushroom [29]. In this research, the major aim was isolation and purification of a novel laccase from *Lepista nuda* and study of its distinctiveness and applications especially its sequence, dye decolorization and its effect on pesticides. These results can help us to better understand the characteristics and functions of *Lepista nuda* laccase, and assess its potential value for future commercialization.

## 2. Results and Discussion

### 2.1. The Classification and Determination of the Isolated Strain LN07

**Figure 1:**
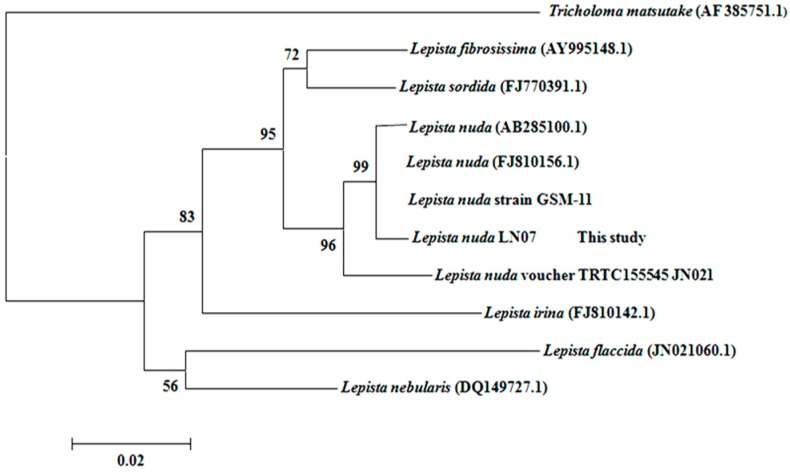
Polymeric analysis of strain LN07 and other tricholomataceae species based on ITS sequences.

Based on the morphological properties of its fruiting body, strain LN07 was initially determined as basidiomycete fungus *Lepista nuda*. After that, a 632-bp fragment of ITS region was amplified and sequenced, and then compared in GenBank using BLAST. The results revealed that strain LN07 was closest to *Lepista nuda* (AB285100.1), *Lepista nuda* (FJ810156.1) and *Lepista nuda* strain GSM-11 with a sequence homology of 99%. A phylogenetic tree on account of ITS region of Tricholomataceae species was constructed, from which we could also see that the strains most closely related to strain LN07 were all *Lepista nuda* (Figure 1). Therefore, strain LN07 was identified as *Lepista nuda* not only from the fruiting body morphology but also from the ITS sequence [19].

### 2.2. Effect of Cu^2+^ Ions on Production of L. nuda Laccase in Liquid Fermentation

**Figure 2:**
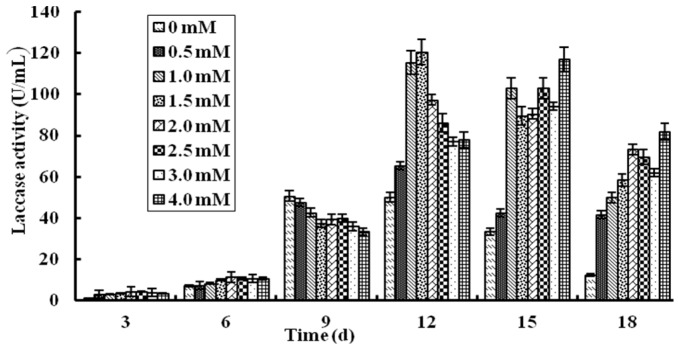
Effects of different concentrations of Cu^2+^ ions on *L. nuda* laccase production in liquid fermentation. Results are presented as mean ± SD (*n* = 3).

Laccases form a group of blue multicopper and polyphenol oxidases and Cu^2+^ ions may affect laccase production [19,30]. In this study, the effect of Cu^2+^ ions on laccase production in liquid fermentation has been proved and the results are shown in Figure 2. Before the sixth day after inoculation, Cu^2+^ ions at different concentrations had virtually no effect on laccase activity and from the ninth day, the laccase activity of all groups began to increase rapidly. when it reached the 12th day, the laccase activity of groups with Cu^2+^ added were all higher than the control group without Cu^2+^ ions and five groups with the Cu^2+^ ion concentration of 0 mM, 0.5 mM, 1.0 mM, 1.5 mM, 2.0 mM respectively attained their highest laccase activities. The group with 1.5 mM Cu^2+^ ions displayed the highest laccase activity of 120 U/mL which was almost triple of the control group. The remaining three groups with the Cu^2+^ ion concentration of 2.5 mM, 3.0 mM and 4.0 mM attained the peak laccase activity 3 days later, but they were all lower than the group with 1.5 mM Cu^2+^. Then, the laccase activity of all groups started to decline. The results manifested that 1.5 mM Cu^2+^ could enhance the laccase activity nearly two-fold compared with the control group and the concentration of Cu^2+^ ions in the group was higher, the peak laccase activity might appear later. This was different from the laccase of *Trametes* sp. LAC-01 which manifested the highest laccase activity in the presence of 2.0 mM Cu^2+^ ions [19].

### 2.3. Purification of L. nuda Laccase

**Figure 3:**
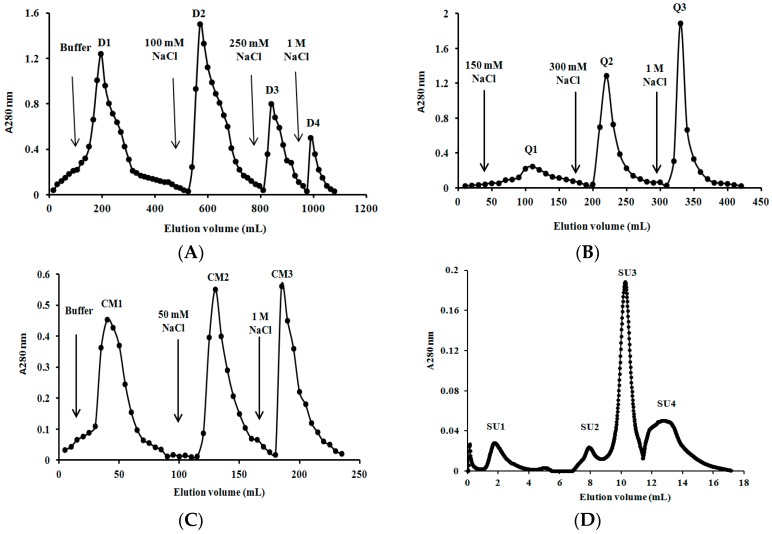
(**A**) Ion exchange chromatography on a DEAE-cellulose column. Sample: Proteins derived from fermentation broth of *Lepista nuda.* Laccase activity resided in fraction D3; (**B**) Ion exchange chromatography on Q-Sepharose column. Sample: Fraction D3 derived from DEAE-cellulose. Laccase activity resided in fraction Q2; (**C**) Ion exchange chromatography on CM-cellulose column. Sample: Fraction Q2 derived from Q-Sepharose. Laccase activity resided in fraction CM2; (**D**) Gel filtration by fast protein liquid chromatography on a Superdex 75 HR 10/30 column using an AKTA Purifier System. Sample: fracton CM2. Flow rate: 0.5 mL per minute. Laccase activity was enriched in fraction SU3.

The crude laccase was chromatographed on DEAE-cellulose, Q-Sepharose and CM-cellulose in succession and three active fractions D3, Q2 and CM2, respectively, were obtained (Figure 3). CM2 fraction was then applied on FPLC and the highest peak (SU3) was the target protein (Figure 3D). The purification process was analogous to that adopted for *Tricholoma mongolicum* laccase [31], but different from protocols employed for *Agrocybe cylindracea* laccase [32] in which SP-Sepharose was utilized instead of CM-cellulose. As summarized in Table 1, laccase from *Lepista nuda* was purified to homogeneity with 185-fold purification resulting in a final specific activity of 163 U/mg and a recovery rate of 35.5%, which were better than the data recorded in other reports [1,31,33].

### 2.4. Determination of Molecular Mass and N-Terminal Amino Acid Sequence of L. nuda Laccase

**Figure 4:**
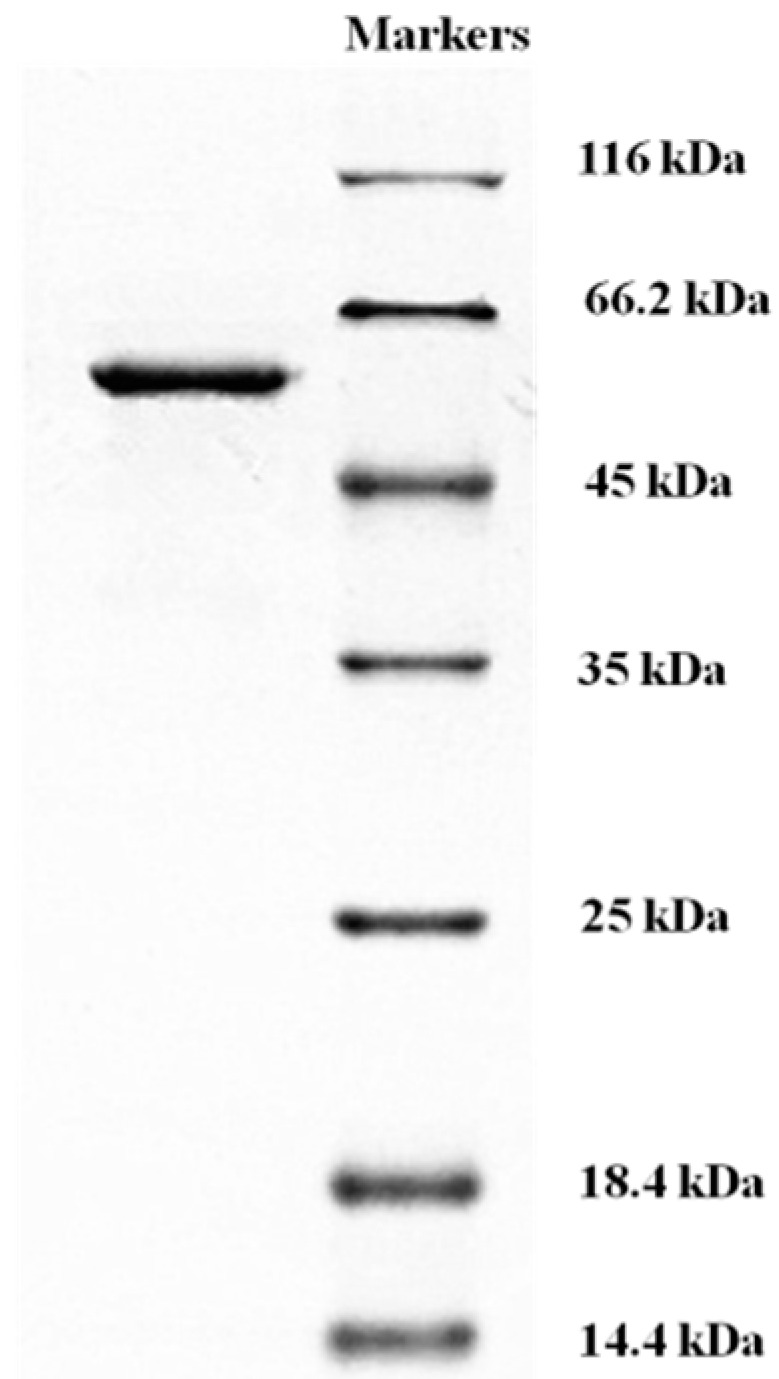
The SDS-PAGE photo of *Lepista nuda* laccase (left lane). Markers were run on the right lane.

*L. nuda* laccase (fraction SU3) appeared as a single band with a molecular mass of 56 kDa in SDS-PAGE (Figure 4) and gel filtration on Superdex 75 yielded the same estimate of molecular mass (Figure 3D), from which we could deduce that *L. nuda* laccase was a monomeric protein with a molecular mass of 56 kDa. The molecular mass of *L. nuda* laccase was within the range of molecular masses for most of the fungal laccases reported (50-100 kDa) [2,9] and similar to *Tricholoma matsutake* laccase (59 kDa) [1] and laccase from *Trametes* sp. LAC-01 (59 kDa) [19] but smaller than *Trametes trogii* laccase (64 kDa) [33].

The N-terminal amino acid sequence of *L. nuda* laccase was AIGPAADLHI VNKDISPDGF which showed the highest homology to *Hypsizygus marmoreus* laccase and only four amino acids were different from *Panus rudis* laccase (Table 2). *L. nuda* laccase manifested considerable homology to those of other fungal laccases (Table 2). Besides, the short signature sequence A I G P was also found in *Russula virescens* laccase [21].

### 2.5. Sequence Determination of L. nuda Laccase by Electrospray Ionization Mass-Mass Spectrometry (ESI-MS/MS) and 2D Nano-Liter Liquid Chromatography & Linear Ion Trap Quadrupole Mass Spectrometer (LC-LTQ)

By ESI-MS/MS, three peptide sequences of the isolated *L. nuda* laccase were obtained. They were RVPNDYNYVSSGAS (lac1), RGGSFSLALNLPVDRG (lac2) and RLPGLVHVSTLGTSVTL (lac3), respectively. There was no homologues sequence in the BLAST/NCBI database detected by blast search. Probably the three peptide sequences represented the unique partial sequences of the purified laccase which can be used to identify the laccase.

Results from LC-LTQ determination showed that five inner peptide sequences EVDSIQIFAGQR (lac4), YSFVLNANQPVDNYWIR (lac5), GINSAILR (lac6), SAGSSVYNYDNPVRR (lac7) and VIEISIPGGTTGFPHPFHLHGHTFDVVR (lac8) were obtained. By blasting in the BLAST/NCBI database, lac4, lac5 and lac6 were highly conserved sequences in the Cu^2+^ combination and conservation zone of laccases and they were highly conserved sequences. Lac7 and lac8 also occurred in the conserved domain of *L. nuda* laccase. Besides, lac8 owned the distinctive sequence (HPFHLHGH) of laccase reported by Giardina *et al.* [2]. 

### 2.6. Effects of pH and Temperature on the Activity of L. nuda Laccase

**Figure 5:**
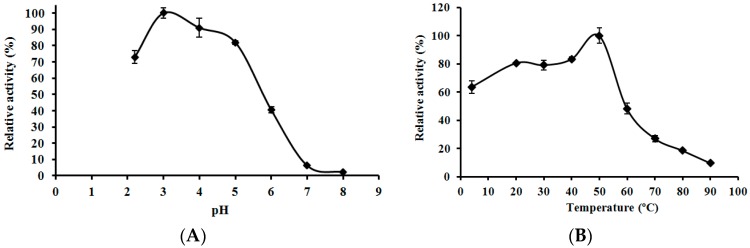
(**A**) Effect of pH on laccase activity of *Lepista nuda* laccase. Results represent mean ± SD (*n* = 3). (**B**) Effect of temperature on laccase activity of *Lepista nuda* laccase. Results represent mean ± SD (*n* = 3).

The optimal pH value of *L. nuda* laccase was about 3.0 which was similar to that of *Clitocybe maxima* laccase [34] but lower than that of *Trametes pubescens* laccase [17]. The relative activity of *L.* *nuda* laccase was above 70% between pH 2.0 and pH 5.0 but there was a sharp decline in enzyme activity as the pH value moved towards 8.0 reaching an almost undetectable level (2.27%) of laccase activity (Figure 5A). The optimal temperature of *L. nuda* laccase was 50 °C, which was the same as that of *Paraconiothyrium variabile* laccase [35]. Above 50% relative activity of *L. nuda* laccase was observed in the temperature range from 4 °C to 55 °C, but the activity dropped rapidly when the temperature reached 60 °C (Figure 5B). 

### 2.7. Effects of Metal Ions and NaCl on Laccase Activity of L. nuda Laccase

The effects of metal ions on *L. nuda* laccase are displayed in Table 3. At the concentration of 1.25 mM, Mg^2+^ and Ca^2+^ ions enhanced the activity of the laccase to 105.9% and 109.3% but Fe^2+^ and Fe^3+^ ions inhibited the laccase strongly with only 28.5% and 14.8% residual relative activity, respectively, different from the case of *Russula virescens* laccase [21]. All the other ions tested inhibited the laccase activity of *L. nuda* laccase to different degrees except K^+^ and Mn^2+^ ions which had little effect on the laccase. *L. nuda* laccase was inhibited by almost all ions as the ion concentration increased. 

Laccases could be inhibited by NaCl which blocked the application of laccase [2,36]. The effect of NaCl on *L. nuda* laccase was carried out and the result showed that the purified laccase endured a certain concentration of NaCl with the half-inhibitory concentration (IC_50_) being 0.2 M (Figure S1), the same as in the case of *Lamprospora wrightii* laccase [37].

### 2.8. Determination of L. nuda Laccase for Kinetic Parameters and Type

Kinetic parameters have been reported for *Tricholoma mongolicum* laccase and *Lamprospora wrightii* laccase [31,37]. According to Michaelis-Menten kinetics, the *Km* and V*max* of *L.* *nuda* laccase were also tested using ABTS as substrate and they were 0.19 mM and 195 μM min^−1^ respectively. The *Km* value of *L. nuda* laccase toward ABTS was higher than those of its counterpart in *Russula virescens* (0.1 mM) [21] and *Paraconiothyrium variabile* (61.7 μM) [35]. It revealed that the affinity of *L.* *nuda* laccase toward ABTS was lower than *Russula virescens* laccase and *Paraconiothyrium variabile* laccase.

According to whether or not laccases possess type I copper, laccases are divided into blue laccases and white laccases [15,16]. No peaks appearing between 600 nm and 620 nm were observed by scanning the spectrum ranging from 190 to 700 nm of the laccase (Figure S2). It could be deduced that the purified *L.* *nuda* laccase was a white laccase similar to *Brassica juncea* laccase [38].

### 2.9. Assay of L. nuda Laccase for HIV-1 Reverse Transcriptase Inhibitory Activity

**Figure 6:**
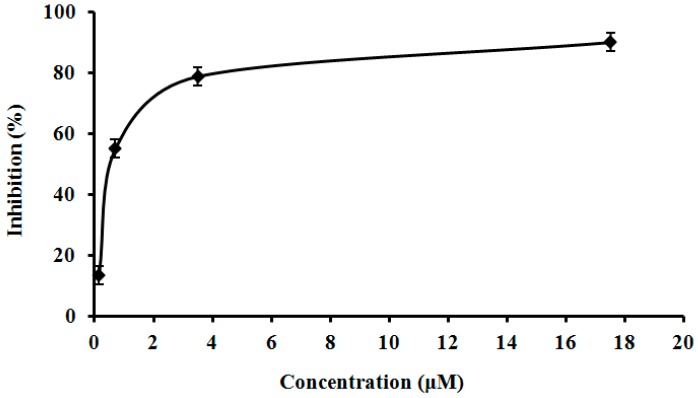
Inhibitory rate of *Lepista nuda* laccase towards to the activity of HIV-1 reverse transcriptase.

The isolated *L.* *nuda* laccase was tested for this activity since some mushroom proteins demonstrated this activity [31,34]. The inhibitory activity of *L.* *nuda* laccase toward HIV-1 reverse transcriptase was dose-dependent. The IC_50_ was 0.65 μM which was apparently lower than that of *Agrocybe cylindracea* laccase [32], indicating that *L.* *nuda* laccase had higher HIV-1 reverse transcriptase inhibitory activity than *A.* *cylindracea* laccase (Figure 6).

### 2.10. The Decolorizing Ability and Pesticide Degradation Ability of the Isolated Laccase

*L.* *nuda* laccase decolorized structurally different dyes with variable decolorization rates (Table 4). Just after incubation for 6 h, almost all laboratory dyes were decolorized, with decolorization above 80% except Methyl orange (67.9%) and crystal violet (21.1%). 88.7% of Bromophenol blue was decolorized after 6 h which was the largest one. The degradation rates of laboratory dyes rose with the increase of incubation time. Compared to previous reports, the ability of decolorize Bromophenol blue and Malachite green of the purified laccase was conspicuously better than *Polyporus brumalis* laccase [39]. Regarding the rate of degradation of Eriochrome black T, *L.* *nuda* laccase was more efficient than laccase from *Trametes* sp. LAC-01 [19] and *Russula virescens* laccase [21]. The data revealed that *L. nuda* laccase demonstrated high decolorizing efficacy towards laboratory dyes. In terms of textile dyes shown in Table 5, Reactive brilliant blue was degraded most rapidly by *L.* *nuda* laccase with the rate of 95.7% after incubation for 6 h and Reactive blue R, Reactive jade blue and Indigo carmine were decolorized with the rates of 71.9%, 64.0% and 51.4%, respectively. However, the rates of decolorization of Reactive brilliant blue and Reactive blue R achieved by *Russula virescens* laccase were just 38% and 32%, considerably lower than the corresponding data for *L*. *nuda* laccase [21]. As the duration of incubation was extended, the rate of decolorization of Indigo carmine increased from 51.4% to 80.4%. It demonstrated that *L.* *nuda* laccase not only decolorized the laboratory dyes but also destained the textile dyes with high degradation rates and had tremendous potential in finding application in the treatment of dye wastewater.

**Figure 7:**
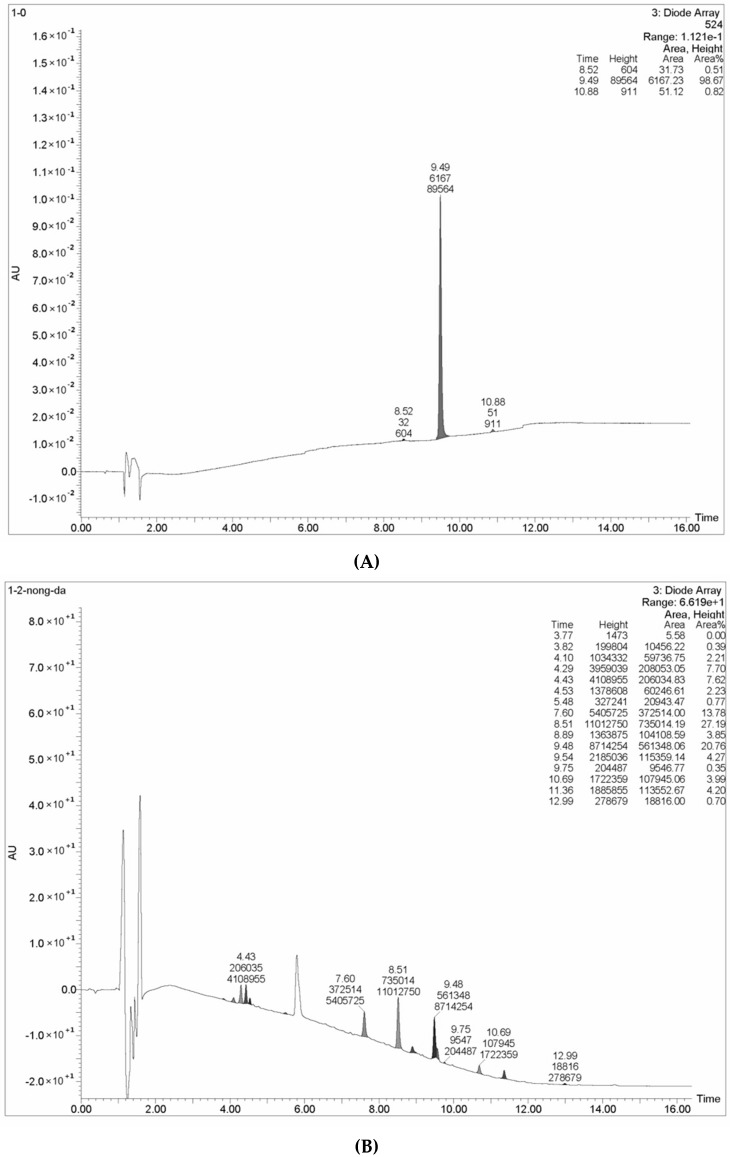
The HPLC chromatogram of methyl red after decolorization by *Lepista nuda* laccase. (**A**) Methyl red control group (**B**) Methyl red which had been subjected to decolorization by the laccase for 48 h.

The HPLC chromatograms of Methyl red reaction products formed following incubation with *L. nuda* laccase for 48 h are shown in Figure 7. In the chromatogram for the control group (Figure 7A), there was a large peak with the retention time of 9.48 which occupied almost the whole height area (98.67%). This represents the spectrum characteristic of Methyl red. Compared to chromatogram for the experimental group (Figure 7B), with addition of *L. nuda* laccase, it showed that more than ten peaks were newly formed with different retention times and the height area of the original peak (with the retention time of 9.48) decreased from 98.67% to 20.76%, indicating that Methyl red was indeed degraded by *L.nuda* laccase and many products or intermediate products were formed. Combined with the mass spectroscopic analysis, some products or intermediate products including benzoic acid, salicylic acid, 2-hydrazinobenzoic acid, phenylhydrazine compound and diphemin appeared, signifying that the N=N double bond and N–C single bond of Methyl red were broken. Although a variety of reports showed that laccase could decolorize dyes, only the decolorization rates of laccase were determined and the degradation products of dyes were not analyzed. These reports show less details than the present investigation [17,21,40]. 

Some pesticides, such as glyphosate [28] and 2,4,6-trichlorophenol [41], could be degraded by laccases. In this study, the pesticide degrading ability of *L.* *nuda* laccase was determined by measuring the absorbance value changes of λ_max_ and the results are presented in Table 5. The absorbance values of λ_max_ of the pestides tested were all altered to some degree with the degradation of alachlor and chlortoluron being most conspicuous. It demonstrated that *L.* *nuda* laccase may degrade pesticides.

## 3. Experimental Section

### 3.1. Strain and Culture Conditions

Strain LN07 was isolated from the fruiting bodies of *Lepista nuda* collected from Yunnan Province of China. Strain identification was based on the morphological properties of its fruiting bodies and a standard ITS sequence amplification and analysis [11]. The mycelia of strain LN07 were cultured on PDA plate culture medium at 26 °C. The submerged cultivation was performed in shaking flasks (120 rpm) containing 500 ml potato dextrose broth and 1.5 mM CuSO_4_ at 26 °C for 12 days and the filtrate was treated as crude laccase.

### 3.2. Assay of Laccase Activity

Laccase activity was determined spectrophotometrically by measuring the oxidation of 2,7-azinobis (3-ethylbenzothia-zolone-6-sulfonic acid) diammonium salt (ABTS). Enzyme solution (10 μL) was mixed with 1 mM ABTS solution (190 μL, in 10 mM sodium acetate buffer, pH 4.5) at 25 °C for 10 min, followed by terminating the reaction with an addition of 10% trichloroacetic acid (300 μL). One enzyme unit (U) was defined as the amount of enzyme required to produce an increase of one absorbance unit at 405 nm per minute per milliliter of the reaction mixture under the assay conditions. All treatments were performed in triplicate [21].

### 3.3. Effect of Cu^2+^ Ions on Laccase Production in Liquid Fermentation 

The effect of Cu^2+^ ions on laccase production in liquid fermentation was tested. CuSO_4_ solution (200 mM) was added to the fermentation medium (potato dextrose broth medium) with the final concentration of 0.5 mM, 1.0 mM, 1.5 mM, 2.0 mM, 2.5 mM, 3.0 mM and 4.0 mM, respectively. The fermentation medium without CuSO_4_ added was treated as control. All experiments were conducted in triplicate. The laccase activity in the fermentation medium was measured at a specified time daily for 3 days after inoculation of strain LN07.

### 3.4. Purification of L. nuda Laccase

After fermentation for 12 days, the liquor was filtered to remove mycelial debris. After centrifugation at 6000 rpm for 30 min, the supernatant was dialyzed in distilled water overnight. Subsequently, an ion exchange chromatography on a column of DEAE-cellulose (2.5 cm × 30 cm, Sigma, St. Louis, MI, USA) previously eluted with 10 mM Tris-HCl buffer (pH 8.6) was carried out at a the flow rate of 2 mL/min. After removal of unadsorbed chromatographic fraction (D1), three adsorbed fractions (D2, D3 and D4) were eluted sequentially with 100 mM NaCl, 250 mM NaCl and 1 M NaCl in the same buffer. All fractions obtained at each purification step were monitored for laccase activity with the enzyme assay mentioned above. Fraction D3 with laccase activity was applied on a Q-Sepharose column (1.5 cm × 20 cm, Bio-Rad, Woodinville, WA, USA). Materials adsorbed on the column were eluted successively with 150 mM NaCl, 300 mM NaCl and 1 M NaCl in 10 mM Trise-HCl buffer (pH 7.2) to yield fractions Q1, Q2 and Q3, respectively. Fraction Q2 containing laccase activity was then purified on a cation exchange column of CM-cellulose (1.5 cm × 20 cm, Sigma). After removing unadsorbed fraction CM1 with 10 mM HAc-NaAc buffer (pH 3.5), adsorbed proteins were desorbed with two gradients of 50 and 1000 mM NaCl in HAc-NaAc buffer resulting in fraction CM2 and fraction CM3. Fraction CM2 with laccase activity was collected after dialysis in distilled water overnight and then subjected to gel filtration by fast protein liquid chromatography (FPLC) (GE Healthcare, Uppsala, Sweden) on a Superdex 75 HR 10/30 column using an AKTA Purifier (GE Healthcare) in 0.2 M NH_4_HCO_3_ buffer (pH 8.5). There were four fractions (SU1, SU2, SU3 and SU4) in the elution profile. SU3 with laccase activity was lyophilized and subsequently used for various assays described below [42,43].

### 3.5. Determination of Laccase Molecular Mass and N-terminal Amino Acid Sequence of L. nuda Laccase 

Sodium dodecyl sulfate-polyacrylamide gel electrophoresis (SDS-PAGE) was performed in accordance with the standard procedure using a 12% resolving gel and a 5% stacking gel to determine the purity and molecular mass of *L. nuda* laccase. Homogeneity was evidenced by the presence of a single band. Electrophoretic mobility of the isolated laccase was compared with those of standard molecular weight marker proteins and the molecular mass was estimated from the calibration curve plotting electrophoretic mobility against molecular mass. In FPLC chromatography, a standard curve based on elution volume and Log *Mr* of molecular mass standards (GE Healthcare) was obtained.

Amino acid sequence analysis was carried out using an HP G1000A Edman degradation unit (Hewlett Packard Company, Palo Alto, CA, USA) and an HP1000 HPLC system (Hewlett Packard Company). Homogeneity was evidenced by the presence of a single amino acid residue in each sequencing cycle [31].

### 3.6. Sequence Determination of L. nuda Laccase by Electrospray Ionization Mass-Mass Spectrometry (ESI-MS/MS) and 2D Nano-Liter Liquid Chromatography & Linear Ion Trap Quadrupole Mass Spectrometer (LC-LTQ) 

After electrophoresis, the single band of *L. nuda* laccase was excised and then eluted from the gel followed by enzymolysis with serine proteinase. Subsequently, electrospray ionization mass-mass spectrometry (ESI-MS/MS) (Michrom BioResources, Auburn, CA, USA) and 2D Nano-Liter Liquid Chromatography (Michrom BioResources) and Linear Ion Trap Quadrupole Mass Spectrometry (Thermo-Fisher, Waltham, MA, USA) were performed to determine some peptide sequences of the laccase. The data were analyzed by the software MaxQuant and Proteome Discoverer [21].

### 3.7. Effects of pH and Temperature on the Activity of L. nuda Laccase

To determine the effect of pH, a series of solutions of ABTS in buffers with different pH values were used. The buffers used were citric acid-Na_2_HPO_4_ buffers (pH range of 2.2–8.0). To determine the effect of temperature, laccase activity was measured at different temperatures ranging from 20 °C to 60 °C instead of 25 °C in the standard assay. The stability of *L. nuda* laccase was also tested at different temperatures (20 °C, 30 °C, 40 °C, 50 °C and 60 °C) for 30 min and 60 min, respectively [19].

### 3.8. Effects of Metal Ions and NaCl on the Activity of L. nuda Laccase 

Different metal ions (Fe^2+^, K^+^, Hg^2+^, Mg^2+^, Pb^2+^, Zn^2+^, Ca^2+^, Cd^2+^, Mn^2+^, Al^3+^ and Fe^3+^) at different concentrations (1.25, 2.5, 5, and 10 mM) were respectively incubated with an equal volume of the purified laccase solution at 4 °C for 1 h. Using the standard assay, the residual laccase activity was measured in triplicate and the control sample was treated with distilled water instead of the solution containing the metal ion tested. The effects of different concentrations of sodium chloride (0 M, 0.2 M, 0.4 M, 0.6 M, 0.8 M, 1.0 M, 1.2 M) on *L. nuda* laccase were quantified using the assay method mentioned above [36].

### 3.9. Determination of L. nuda Laccase for Kinetic Parameters and Type

Kinetic studies of the purified laccase were performed using ABTS as substrate in a series of concentrations ranging from 0.0625 mM to 2.0 mM in the HAc–NaAc buffer (pH 4.6) at 25 °C. The reciprocals of the substrate concentrations and the reciprocals of the corresponding initial velocities were then used to generate the Lineweaver-Burk plot.

The laccase type that *L. nuda* laccase belongs to was determined by scanning the absorption spectrum at wavelengths ranging from 190 to 700 nm. The presence of an absorption peak between 600 nm and 620 nm would indicate that the laccase is a blue laccase. Otherwise, it is a white laccase [16,17].

### 3.10. Assay of L. nuda Laccase for HIV-1 Reverse Transcriptase Inhibitory Activity

The assay of HIV-1 reverse transcriptase (RT) inhibitory activity was performed following instructions supplied with the enzyme-linked immunosorbent assay kit from Boehringer Mannheim (Mannheim, Germany). The assay takes advantage of the ability of RT to synthesize DNA, starting from the template per primer hybrid poly(A) oligo(dT)_15_. The digoxigenin- and biotin-labeled nucleotides are incorporated into one of the same DNA molecules in an optimized ratio, which is freshly synthesized by the RT. The detection and quantification of synthesized DNA as a parameter for RT activity follows a sandwich ELISA protocol. Biotin-labeled DNA binds to the surface of microtiter platemodules that have been precoated with streptavidin. Subsequently, an antibody to digoxigenin, conjugated to peroxidase (anti-DIG-POD) binds to the digoxigenin-labeled DNA. Finally, the peroxidase substrate is added. The peroxidase catalyzes the cleavage of the substrate and produces a colored reaction product. The absorbance of the samples at 405 nm can be determined by a microtiter plate (ELISA) reader and is directly correlated to the level of RT activity. A fixed amount (4–6 ng) of recombinant HIV-1 RT was used. The inhibitory activity of *L. nuda* laccase was calculated as percent inhibition as compared to a control without *L. nuda* laccase [31].

### 3.11. Decolorizing and Pesticide Degrading Abilities of L. nuda Laccase

Some common laboratory dyes such as Methyl red, Methyl orange, Eriochrome black T, Coomassie brilliant blue, Crystal violet, bromophenol blue and Malachite green were purchased from Sigma. Some reactive dyes used in the textile industry including Reactive blue R, Reactive brilliant blue, Reactive jade blue, Reactive red, Reactive brilliant orange, Reactive black and Indigo carmine were bought from the number five chemical factory in the city of Zhangjiagang in China. The aforementioned dyes were used to evaluate the ability of the purified laccase to decolorize dyes. Different concentrations of the dyes were incubated at 37 °C in 400 mL 10 mM sodium acetate buffer (pH 4.6) containing 10 mL *L. nuda* laccase solution for 6 h, 12 h, 24 h and 96 h respectively. In parallel, a negative control which contained all components except *L. nuda* laccase was incubated in triplicate. The decolorizing ability of *L. nuda* laccase was determined spectrophotometrically as the relative decrease of the absorbance of dyes at their maximum absorption wavelength. The maximum absorption wavelengths of the dyes are shown in Table 4. In order to analyze the effect of laccase on degradation of Methyl red whose structure is relatively simple, the above mentioned reaction system was incubated for 48 h but with a scale 10 times larger. Then the reaction products formed from Methyl red were subjected to liquid chromatography-mass spectrometry (LC-MS) analysis. The control group was treated in the same way as described above but in the absence of *L. nuda* laccase.

The method of determining the pesticide degrading ability of *L. nuda* laccase was similar to the method for dyes but different pesticides were used instead of dyes. Incubation was carried out for 3 h, 6 h, 12 h and 24 h, respectively. The pesticides tested comprised glyphosate, pyrimethanil, quizalofop-P, chlortoluron, diuron, alachlor, prometryn and simazine. All treatments were performed in triplicate and the control group was treated in the same way as described above but in the absence of *L. nuda* laccase [21].

## 4. Conclusions

In conclusion, a monomeric laccase with a molecular mass of 56 kDa was purified and characterized from *Lepista nuda*. It was a white laccase without type I copper which was similar to *Brassica juncea* laccase [38]. Its N-terminal amino acid sequence was AIGPAADLHIVNKDISPDGF and eight inner peptide sequences were determined among which lac4, lac5 and lac6 sequences were highly conserved and just in the Cu^2+^ combination and conservation zones of laccases. *Lepista nuda* laccase had higher HIV-1 reverse transcriptase inhibitory activity than the *Agrocybe cylindracea* laccase [32] and maybe it could be treated as a drug source of AIDS treatment. After fermentation for 12 days, 1.5 mM Cu^2+^ ions could enhanced the laccase activity nearly two-fold compared with the control group. The optimal pH value and temperature were pH 3.0 and 50 °C, respectively and its thermostability was relatively high. The laccase activity was enhanced by Mg^2+^ and Ca^2+^ ions but inhibited by other ions used in this study. The purified laccase was inhibited by NaCl with an IC_50_ of 0.2 M. Using ABTS as substrate, the Km and Vmax of the laccase were, respectively, 0.19 mM and 195 μM. Several dyes including laboratory dyes and textile dyes were structurally degraded by the laccase especially Methyl red that was demonstrated by LC-MS analysis. Besides, the laccase affected the absorbance at the maximum wavelength of many pesticides. Thus, *Lepista nuda* laccase had potential value for commercialization especially for industrial and environmental applications such as textile finishing and wastewater treatment.

## Figures and Tables

**Table 1:** Yields and laccase activities of various chromatographic laccase-enriched fractions from *Lepista nuda* laccase (assay conducted at 25 °C).

Fraction	Total Protein (mg)	Specific Laccase Activity (U/mg)	Total laccase ACTIVITY (U)	Recovery of Laccase Activity (%)	Purification Fold
Crude	1307.6	0.9	1149.4	100.0	1.0
D3	241.2	3.3	802.1	69.8	3.8
Q2	25.3	26.2	662.8	57.7	29.8
CM2	9.2	50.5	464.8	40.4	57.4
SU3	2.5	163.3	408.3	35.5	185.8

**Table 2:** Comparison of the N-terminal amino-acid sequences of *Lepista nuda* laccase and other fungal laccases.

Accession Number	Fungus	N-Terminal Amino Acid Sequence
This study	*Lepista nuda*	1 A I G P A A D L H I V N K D I S P D G F 20
AFN10626.1	*Hypsizygus marmoreus*	20 I G P S A D M H V V N K D I S P D G F 38
AFD97049.1	*Coprinus comatus*	22 A I G P N A D L F I V N K D I A P D G F 41
AFD97050.1	*Coprinus comatus*	19 A I G P V A D L H I V N R I I A P D G F 38
AFV15785.1	*Leucoagaricus gongylophorus*	26 I G P T S D M Y I V N K D I S P D G F 44
AAR13230.1	*Panus rudis*	1 A I G P V T D L H I V N D N I A P D G F 20
AAW28937.1	*Trametes* sp. 420	25 A I G P V T D L N I V N A N I S P D G F 44
ADD14077.1	*Pleurotus eryngii*	24 A I G P I A D M Y I V N E D V S P D G F 43
AAR82932.1	*Pleurotus ostreatus*	24 A I G P T G D M Y I V N E D V S P D G F 43
AAR03582.1	*Volvariella volvacea*	19 A I G P V T E L Q I V N D E I A P D G F 38
BAJ12090.1	*Lentinuda edodes*	20 A I G P V T D L H V V N K F I Q P D G F 39
AAR21094.1	*Pleurotus ostreatus*	24 A I G P T G N M Y I V N E D V S P D G F 43

Identical residues are shaded.

**Table 3:** Effects of various metal ions on laccase activity of *Lepista nuda* laccase.

Metal Ions	Relative Laccase Activity (% of Control)			
	10 mM	5 mM	2.5 mM	1.25 mM
Fe^2+^	8.4 ± 3.2	14.8 ± 0.5	20.6 ± 0.5	28.5 ± 1.2
K^+^	85.1 ± 2.1	87.7 ± 3.9	92.1 ± 3.9	94.1 ± 3.1
Hg^2+^	41.1 ± 1.7	58.9 ± 3.8	64.3 ± 2.7	68.9 ± 2.3
Mg^2**+**^	91.1 ± 3.7	101.2 ± 4.2	108.9 ± 3.9	105.9 ± 3.6
Pb^2+^	72.0 ± 3.1	77.2 ± 2.9	78.4 ± 2.5	71.7 ± 2.6
Zn^2+^	35.1 ± 2.1	53.0 ± 2.5	65.7 ± 2.6	67.6 ± 2.2
**Ca^2+^**	90.3 ± 4.3	100.2 ± 3.6	105.2 ± 0.9	109.3 ± 3.6
Cd^2+^	58.6 ± 2.1	60.7 ± 2.3	83.7 ± 3.1	75.4 ± 1.3
Mn^2+^	81.9 ± 4.7	83.7 ± 3.2	81.7 ± 3.2	91.5 ± 2.7
Al^3+^	60.8 ± 1.4	67.8 ± 2.1	70.4 ± 2.6	84.4 ± 2.9
Fe^3+^	16.1 ± 0.9	1.9 ± 0.3	6.29 ± 2.2	14.8 ± 0.7

Results represent mean ± SD (*n* = 3). Laccase activity in the absence of metal ions was regarded as 100%.

**Table 4:** Decolorization of different dyes after incubation with *Lepista nuda* laccase for different durations.

Dyes	λ_max_	Concentration	Decolorization (%)			
	(nm)	(mg/L)	6 h	12 h	24 h	96 h
Methyl red	524	250.0	82.3	81.5	83.2	83.5
Methyl orange	460	12.5	67.9	70.3	71.1	74.3
Eriochrome black T	540	125.0	81.0	81.1	83.4	84.9
Coomassie brilliant blue	549	25.0	80.7	86.0	87.5	86.6
Crystal violet	584	5.0	21.1	22.0	28.0	32.2
Bromophenol blue	590	25.0	88.7	90.0	90.3	91.1
Malachite green	614	6.3	83.0	86.7	86.0	86.8
Reactive brilliant orange	492	50.0	0.3	1.6	1.9	1.4
Reactive red	546	50.0	0.4	0.5	0.6	0.9
Reactive black	585	50.0	4.8	3.3	4.8	4.9
Reactive blue R	592	125.0	71.9	71.0	72.3	72.5
Reactive brilliant blue	605	100.0	95.7	94.9	95.8	95.0
Reactive jade blue	627	50.0	64.0	66.8	70.7	75.4
Indigo carmine	609	25.0	51.4	66.3	78.3	80.4

**Table 5:** The pesticide-degrading effect of *Lepista nuda* laccase on various pesticides.

Pesticides	λ_max_	Concentration	|Changes of OD (%)|			
	(nm)	(mg/L or *w*/*w*)	3 h	6 h	12 h	24 h
glyphosate	279	1.0 mg/L	16.4	15.8	18.6	24.1
pyrimethanil	268	0.3 mg/L	21.9	40.1	51.6	55.3
quizalofop-P	224	0.03%	136.0	154.7	170.5	198.5
chlortoluron	248	1.0%	224.0	234.9	248.0	240.6
diuron	266	0.03%	17.0	23.2	29.6	41.1
alachlor	203	0.01%	235.4	327.8	464.5	564.4
prometryn	266	0.03%	28.1	34.5	30.1	34.6
simazine	267	0.05%	26.2	25.6	31.8	37.6

|| means the absolute value.

## Supplementary Materials

Supplementary materials can be accessed at: http://www.mdpi.com/1420-3049/21/4/415/s1.
