# Supplementary material for: Purification and Characterization of a White Laccase with Pronounced Dye Decolorizing Ability and HIV-1 Reverse Transcriptase Inhibitory Activity from Lepista nuda
Source: Molecules. 2016 Mar 26;21(4):415. doi: 10.3390/molecules21040415 (PMC6274495; doi:10.3390/molecules21040415)
Supplement: Supplementary file 1 [file molecules-21-00415-s001.pdf]

Supplementary Materials: Purification and Characterization of a White Laccase with Pronounced Dye Decolorizing Ability and HIV-1 Reverse Transcriptase Inhibitory Activity from *Lepista nuda*

Mengjuan Zhu, Guoqing Zhang, Li Meng, Hexiang Wang, Kexiang Gao and Tb Ng

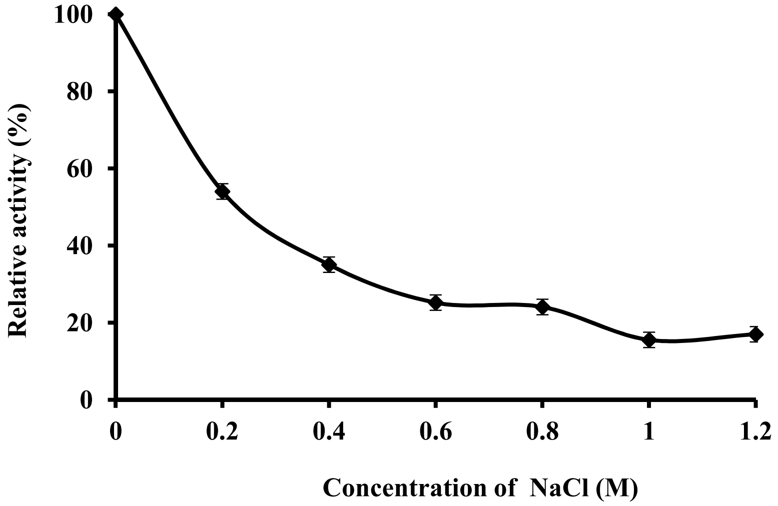

Figure S1. The tolerance of *Lepista nuda* laccase to NaCl.

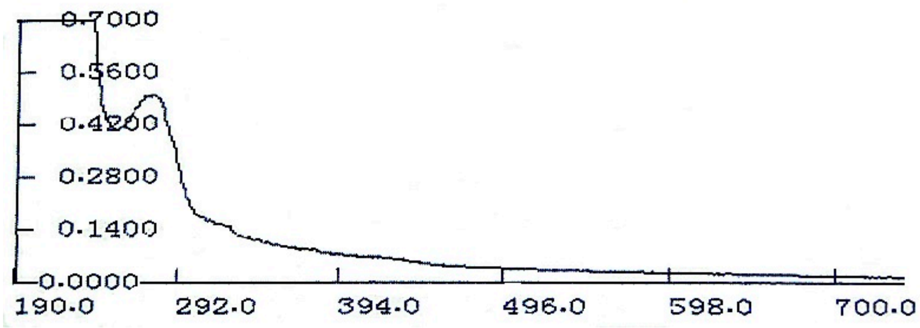

Figure S2. The spectrum scanning map of *Lepista nuda* laccase. The wavelength was from 190 nm to 700 nm.
